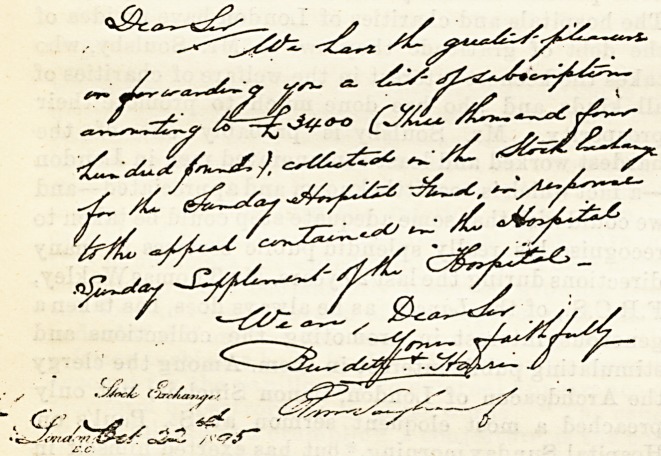# The Metropolitan Hospital Sunday Fund, 1895

**Published:** 1895-10-26

**Authors:** 


### THE METROPOLITAN HOSPITAL SUNDAY FUND, 1895.

### UPWARDS OF £60,000 RECEIVED—£18,000 FROM "THE HOSPITAL'S" APPEAL.

For years it has been the hope of many connected with the Sunday Fund that at least £50,000 might be ultimately raised in connection with the Fund. The largest sum ever received until the present year was £45,331 in 1891, when the late Duke of Cleveland gave £5,000, and £1,000 was by Sir Savile Crossley, Bart., who has given a similar sum every year since to the Fund. Of the £60,000 received this year, upwards of £20,000 has been paid to the Lord Mayor, at the Mansion House, by individual donors and others, who have given in this way in addition to those who contributed through the churches. It is important to remember that every penny of the money sent to the Lord Mayor goes direct to the hospitals without practically any deduction for expenses, and that the working expenses of the Sunday Fund amount on an average to only 3½ per cent., whilst this year these expenses will not, we expect, exceed 2½ per cent., if they even amount to as much as that.

### How the Money has been Raised.

The final effort so willingly made by the members of the Stock Exchange at the invitation of Messrs. Burdett and Harris, and Messrs. Pim, Vaughan, and Co., who have both worked enthusiastically, is typical of the movement which has this year placed the Hospital Sunday Fund collections upon a new and solid basis. Any one who refers to the list of individual contributions, which we gladly publish by request in another column (page 71), will see that the movement represents every section of opinion, for all have felt it to be a privilege to help the hospitals in their present difficulty, and thus a gratifying success has been secured. The members of the Stock Exchange are amongst the most generous of men, and have this year given to St. Thomas's and other hospitals, apart from handsome individual contributions sent direct to charities, upwards of £6,000. In the same way, residents throughout London have shown an interest in this year's movement, and the authorities of the Fund inform us that the efforts of The Hospital have resulted in the large sum of upwards of £18,000 being sent to the Lord Mayor in direct response to the appeal made in our special Hospital Sunday Supplement. We are conscious that our efforts would have failed had we not received the united co-operation of our colleagues in the metropolitan Press. Never before, in the history of the Fund, has the Press taken such concerted action on behalf of the hospitals. The *Times*, for the first time in its history, consented to insert and give prominence to the blocks contained in our article entitled "A Word to Living Londoners," took a step which had a material influence upon the amounts collected in the churches, and no doubt helped immensely to quicken public interest, and so to contribute to the success of the effort. The *Standard*, the *Daily News*, the *Daily Chronicle*, the *Morning Post*, the *Daily Graphic*, the *Morning Advertiser*, the *Pall Mall*, the *St. James's*, and *Westminster Gazettes*, the *Globe*, and the Sunday and weekly papers, all cordially co-operated; whilst, last but not least, the *Lancet* issued once more a special supplement, which was widely circulated. The victory is, therefore, a journalistic victory, and should encourage the Press, as we hope it will do, to continue the effort year by year, so as to provide that the Metropolitan Hospital Sunday Fund shall never again yield less than £50,000. The *Daily Telegraph* was too much occupied with the Grace Fund to be able to help this year, but no doubt next year we may rely upon its hearty co-operation.

### Thanks Especially Due.

The Lord Mayor, Sir Joseph Renals, Bart., has displayed a keen interest in the success of the Hospital Sunday Fund during his mayoralty, and has made more than one effort to draw public attention to the necessity of its being placed year by year on a basis commensurate on the one hand to the needs of the hospitals, and on the other to the wealth of the citizens of London. In this connection we would specially thank Mr. W. J. Soulsby, who for twenty years has been private secretary to successive Lord Mayors. The hospitals and charities of London have no idea of the debt of gratitude they owe to Mr. Soulsby, who takes the keenest interest in the welfare of charities of all kinds, and who has done much to promote their prosperity. Mr. Soulsby is probably one of the hardest worked and least remunerated men in London —a fact which is too little known and appreciated—and we could wish that some adequate step could be taken to recognise his really splendid public services in many directions during the last 20 years. Mr. Thomas Wakley, F.R.C.S., of the *Lancet*, as he always does, has taken a generous interest in promoting the collections and stimulating public interest in them. Among the clergy the Archdeacon of London, Canon Sinclair, not only preached a most eloquent sermon at St. Paul's on Hospital Sunday morning,* but has exerted himself in many ways to raise the Fund to £50,000 this year. Many other preachers, including the Archbishop of Canterbury, the Dean of Westminster, Dean Farrar, Canons Ainger, Fleming, Ridgeway, Wilberforce, and Keatinge, the Chief Rabbi, and many others, have united with the Press to secure this splendid result, which will make the Hospital Sunday collections in 1895 memorable for all time. The success proves what can be done by united effort, and we hope that now the Fund has at last exceeded £50,000, it will never again amount to less than that sum. Both preachers and writers were materially aided by the statistics, prepared for publication with great care by Mr. R. A. Owthwaite, the Fund's statistician, under the direction of the able secretary, Mr. Henry N. Custance, to both of whom grateful acknowledgments are due.

### Letters Received.

It may interest some of our readers to reproduce

* Since published by the Scientific Press, 428, Strand, W.C., and should be secured by all interested in our hospitals.

two of the letters we have received containing cheques for the Hospital Sunday Fund this year.

A Cheque for £10,000.

The following letter is from Mr. Daniel Marks, of Messrs. Marks, Bulteel, Mills, and Co., one of the most generous and kindly of men :—

To the Editor,
"The Hospital".

Sir,

HOSPITAL SUNDAY 1895.

I have much pleasure in informing you, on behalf of Mr B. J. Barnato and other City friends, that we have forwarded to the Lord Mayor £10,000 in response to the appeal contained in the illustrated Hospital Sunday Supplement of "The Hospital" towards the £70,000 required to meet the present needs of the whole of the voluntary hospitals of London.

I am, Sir,

Your obedient Servant,

Daniel Marks

A Cheque for £3,400.

The following letter is from the two firms who worked so zealously and successfully in obtaining subscriptions from the members of the Stock Exchange :—

## Figures and Tables

**Figure f1:**
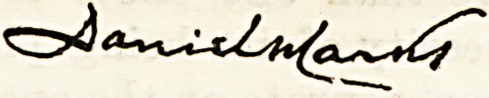


**Figure f2:**